# The Role of Al/Ti in Precipitate-Strengthened and Austenite-Toughened Co-Free Maraging Stainless Steel

**DOI:** 10.3390/ma17215337

**Published:** 2024-10-31

**Authors:** Qihan Meng, Shuai Tian, Zhenbao Liu, Xiaohui Wang, Wenyu Zhao, Changjun Wang, Yongqing Sun, Jianxiong Liang, Zhiyong Yang, Jinli Xie

**Affiliations:** 1 Institute for Special Steel Institute, Central Iron and Steel Research Institute, Beijing 100081, China; 18706809561@163.com (Q.M.); tianshuai@nercast.com (S.T.); wangxiaohui@nercast.com (X.W.); zhaowenyu@nercast.com (W.Z.); wangchangjun@nercast.com (C.W.); sunyongqing@nercast.com (Y.S.); liangjianxiong@nercast.com (J.L.); yangzhiyong@nercast.com (Z.Y.); 2 Gaona Aero Material Co., Ltd., Beijing 100081, China

**Keywords:** maraging stainless steel, strength, precipitates, austenite

## Abstract

The strength of ultra-low carbon maraging stainless steels can be significantly enhanced by precipitating nanoscale intermetallic secondary phases. Retained or reversed austenite in the steel can improve its toughness, which is key to achieving an ideal combination of strength and toughness. Ti and Al are often used as cost-effective strengthening elements in maraging stainless steels but the synergistic toughening and strengthening mechanisms of Ti and Al have not been studied. To investigate the synergistic toughening and strengthening mechanisms of Ti and Al in Co-free maraging stainless steels, this paper focuses on the microstructure and mechanical properties of three alloys: Fe-12Cr-11Ni-1.7Al-0.5Ti (Steel A), Fe-12Cr-11Ni-0.5Ti (Steel B), and Fe-12Cr-11Ni-1.7Al (Steel C). The impact of Ti and Al on the microstructure and mechanical properties was investigated using X-ray diffraction (XRD), high-resolution transmission electron microscopy (TEM), and thermodynamic simulations. The relationship between microstructure, strength, and toughness is also discussed. The results indicated that Steel A, containing both Al and Ti, exhibited the highest strength level after solution treatment at 900 °C, with an ultimate tensile strength reaching 1571 MPa after aging at 540 °C. This is attributed to the simultaneous precipitation of spherical β-NiAl and rod-shaped η-Ni_3_Ti phases. Steel B, with only Ti, formed a significant amount of Ni-rich reversed austenite during aging, reducing its ultimate tensile strength to 1096 MPa. Steel C, with only Al, showed a high strength–toughness combination, which was achieved by forming dispersive nano-sized intermetallic precipitates of β-NiAl in the martensitic matrix with a slight amount of austenite. It is highlighted that Al has superior toughening and strengthening effects compared to Ti in the alloy system.

## 1. Introduction

Since its inception in the 1940s, high-strength stainless steel has evolved through four generations, with its strength reaching 2.1 GPa [1]. High-strength stainless steels possess ultra-high strength, excellent ductility, and corrosion resistance, making them widely used in aerospace, ocean engineering, and petrochemical industries [2]. Maraging stainless steels, based on the Fe-Cr-Ni alloy system, typically have a carbon content of less than 0.03%, such as the typical steel grades PH13-8Mo and Custom465. The microstructure of these steels at room temperature includes lath martensite, a small amount of austenite, and strengthening phases. In maraging stainless steels, austenite plays an important role in toughening, which comes from two sources: austenite retained from an incomplete transformation after martensitic transformation during solution treatment and reversed austenite precipitated during aging at the boundaries of martensite laths or original austenite grain boundaries [3,4,5,6]. If the temperature is further reduced after the solution treatment, austenite will continue to transform into martensite. Both retained and reversed austenite can alleviate stress concentration at crack tips, thereby enhancing the toughness of a material [7]. Maraging stainless steel is primarily strengthened by the precipitation of strengthening phases on the high-dislocation-density lath martensite used to pin dislocations [8]. In different alloy systems, the types of strengthening phases vary but they can generally be divided into three categories: carbides (such as MC and M_2_C), intermetallic compounds (such as NiAl and Ni_3_Ti), and element-enriched phases (such as epsilon phase and alpha prime phase). The strengthening effect depends on the size, number density, volume fraction, and spatial distribution of the strengthening phases. Due to the increasing demand for high-strength stainless steel in the equipment manufacturing industry, there is a greater demand for cost-effectiveness, especially for high-strength stainless steels containing expensive precious metals, such as Co, Mo, and Nb. Al and Ti are more abundant and less expensive compared to the aforementioned traditional strengthening elements. This makes Al and Ti attractive options for reducing the overall cost of high-strength stainless steels without compromising their performance. Additionally, both Al and Ti contribute to the precipitation strengthening of stainless steels. Al forms intermetallic phases, such as β-NiAl, which precipitates as spherical nanoparticles in the martensite matrix, providing excellent strengthening effects due to its coherence with the matrix. Ti forms rod-shaped η-Ni_3_Ti phases, which contribute to the strength through a combination of precipitation strengthening and grain boundary pinning mechanisms. Therefore, many scholars have investigated low-cost alloy systems and the development of new alloy systems with the addition of Ti, Al, and other inexpensive metals has become a popular research topic, as shown in Table 1. High-strength stainless steels, such as MLX17 and MLX19, were included in the MMPDS manual in 2017 and future research efforts will likely continue to pursue the development of cheaper alloys with a higher strength.

Many previous studies have reported the strengthening mechanisms of Al and Ti in maraging steels. For instance, Charline et al. [10] discovered that maraging steels containing Al were strengthened through the precipitation of the B2 ordered phase β-NiAl, which has a lattice parameter nearly identical to that of body-centered cubic (BCC)-Fe. This similar structure allowed it to be uniformly distributed within the matrix as spherical nanoparticles, thereby providing excellent strengthening effects. In Ti-containing maraging steels, Ti precipitates as a rod-shaped η-Ni_3_Ti phase with a close-packed hexagonal crystal structure [11], exhibiting an orientation relationship with the matrix of (110)α∥(0001)η and [111]α∥[1120]η [12]. Via strengthening by intermetallic compounds, such as NiAl and Ni_3_Ti, the strength of maraging steels can be increased to over 1.5 GPa [13,14,15]. The ultimate tensile strength of MLX19 alloy approached 1.9 GPa due to the synergistic strengthening by the nanoscale intermetallic compounds NiAl and Ni_3_Ti [16,17,18,19]. Leitner et al. [20] investigated how adding Ti to PH13-8Mo alloy affected its mechanical properties. The results indicated that PH13-8Mo alloy without added Ti precipitated in an ordered spherical NiAl phase uniformly throughout the martensitic matrix rich in solute clusters. The PH13-8Mo alloy with added Ti precipitated in a rod-shaped hexagonal Ni_3_Ti phase.

The formation of intermetallic phases of NiAl or Ni_3_Ti through Al and Ti in the alloy increased the strength but the toughening effect of austenite cannot be overlooked because a large amount of austenite can reduce the yield strength of the material. The current research gap in the field of maraging stainless steels highlights the need for a comprehensive study on the synergistic effects of Al and Ti on both precipitation strengthening and austenite toughening. Therefore, based on the alloy composition of Fe-12Cr-11Ni-1.7Al-0.5Ti (wt.%), this article reports the microstructure and mechanical properties of three types of alloys with Al, Ti, and Al/Ti. The influence of alloying elements on the precipitation of the second phase and austenite is explored and the strengthening and toughening mechanisms of Al and Ti in steel are also revealed here. Additionally, the quantification of the mechanical properties was provided, particularly the ultimate tensile strength and impact absorption energy, which are critical for applications requiring a balance of strength and toughness. The provision of a theoretical framework and empirical data can guide the design and development of new Co-free maraging stainless steels with optimized compositions for enhanced performance. This study not only advances the understanding of the role of Al and Ti in maraging stainless steels but also contributes to the development of more cost-effective and high-performance materials for various industrial applications.

## 2. Materials and Methods

**Figure 1:**
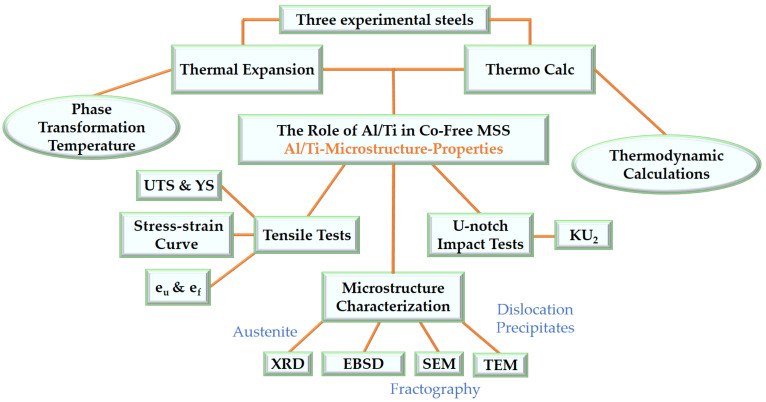
Methodological diagram. UTS = ultimate tensile strength, YS = yield strength, KU_2_ = U-notch impact absorption energy, e_u_ = uniform elongation, e_f_ = elongation at fracture.

The methodological diagram (Figure 1) provides a description of the steps taken to develop the research. Alloy ingots were melted using a 50 kg vacuum induction furnace, heated to 1180 °C, and then forged into round bars with a diameter of 15 mm. The composition of the alloys is shown in Table 2. Steel A was a stainless alloy containing both Al and Ti, Steel B contained only Ti, and Steel C contained only Al, with other elements, such as C, Cr, Ni, and Mo, being essentially at the same levels as those in Steel A.

**Figure 2:**
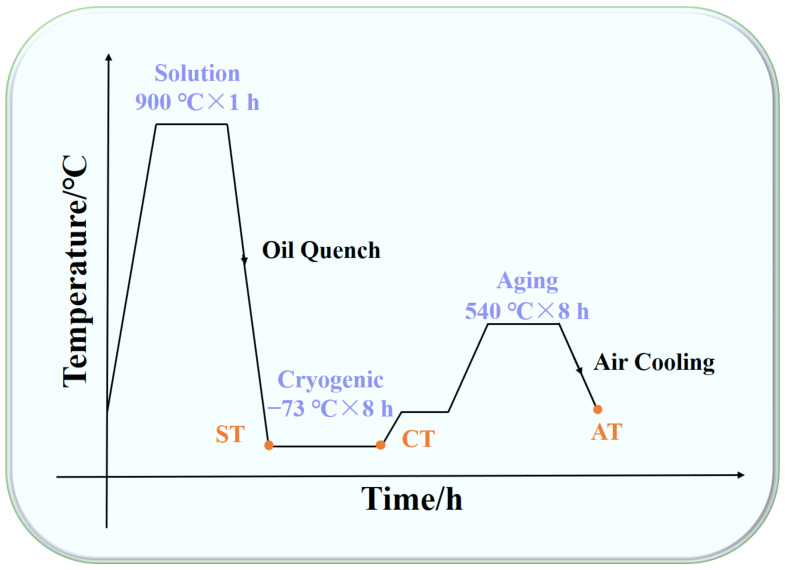
Heat treatment routes for experimental steels.

The phase transformation temperatures of the three alloys were determined using a DIL 805-type dilatometer. The samples were heated at a fixed rate of 200 °C/h to 1000 °C and held for 5 min then cooled to −100 °C at a fixed rate of 5 °C/s. The specimens of the three alloys were all solution-treated at 900 °C (ST), held for 1 h, then oil quenched to room temperature. This was followed by deep cryogenic treatment (CT) at −73 °C for 8 h before returning to room temperature and being aged at 540 °C for 8 h (AT). The samples in the solution, deep cryogenic, and aging conditions were named ST, CT, and AT, respectively (Figure 2). The bar tensile and impact specimens were processed and tested according to the requirements of the Chinese national standards GB/T 228.1-2021 and GB/T 229-2020 after the heat treatment. Room-temperature tensile tests and U-notch impact tests were conducted on a universal material testing machine (MTS-880, MTS Corporation, Woodbury, MN, USA) and an impact testing machine(Ni300, NCS Testing Technology Corporation, Beijing, China), respectively. The experimental data were taken as the average of three tested samples.

The aged samples were prepared into circular specimens with a diameter of 3 mm and a thickness of 70 μm and electropolished under a dual-jet stream at 18 V and 20 °C using an electrolyte of 6% HClO_4_ and 94% C_2_H_5_OH (volume fraction) to obtain TEM samples. High-resolution transmission electron microscopy (HRTEM) was performed on a FEI Tecnai G2 F20. Samples for electron backscatter diffraction (EBSD) tests were electropolished in a mixed solution of 10% HClO_4_ and 90% C_2_H_5_OH and the EBSD data were analyzed using the HKL Channel5 2019 v5.12 software package. Specimens with dimensions of 10 × 10 × 20 mm were polished and subjected to X-ray diffraction (XRD) using a Co-Kα target to determine the phase fraction of austenite. The scanning step was 0.02°, the scanning speed was 76.8°/min, and the 2θ scanning range was 45–115°. The XRD patterns were analyzed and processed using MDI Jade 6.0 software and the volume fraction of austenite and dislocation density were calculated based on the diffraction data. The fracture morphology was characterized on a Tescan MIRA 3 XMH field-emission scanning electron microscope (SEM).

## 3. Results and Discussion

### 3.1. Thermal Expansion Analysis and Thermodynamic Calculations

**Figure 3:**
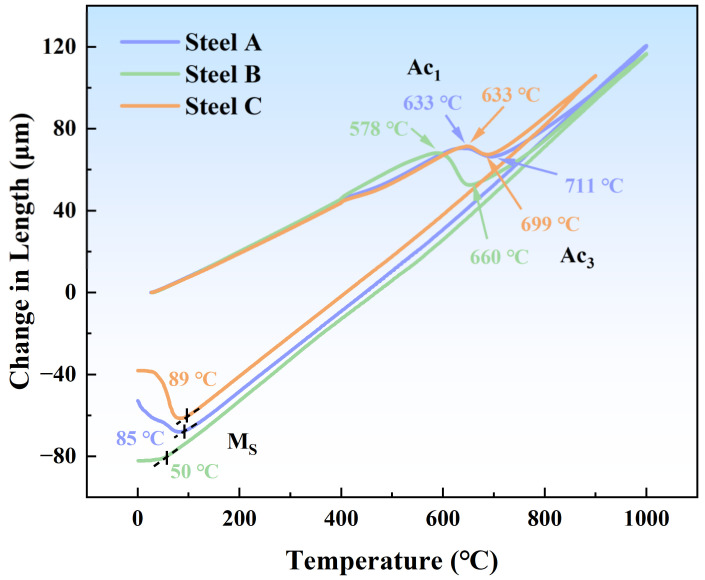
Dilatometry curves of experimental steels.

The dilatometry curves of the three alloys after heating and cooling are depicted in Figure 3. Steel B exhibited the lowest martensitic transformation temperature (Ms) at 50 °C while Steel A and Steel C showed Ms temperatures of 85 °C and 89 °C, respectively. Empirical relationships from the literature between alloying elements and the Ms of maraging stainless steel are given by the formula [21]: Ms(°C) = 550 − 330C − 35Mn − 17Ni − 12Cr − 21Mo − 10Cu − 5W − 10Si − 0Ti + 10Co + 30Al (where element values are given in wt.%, with a margin of error of ±20 °C). Al significantly raised the Ms temperature, whereas Ti had a minimal impact. Steel B, which lacked Al, had the lowest Ms temperature (Figure 3) and Steel A, which contained both Al and Ti, had an intermediate Ms temperature. This suggests that within the range of alloy compositions studied in this paper, Ti reduced the Ms temperature.

**Figure 4:**
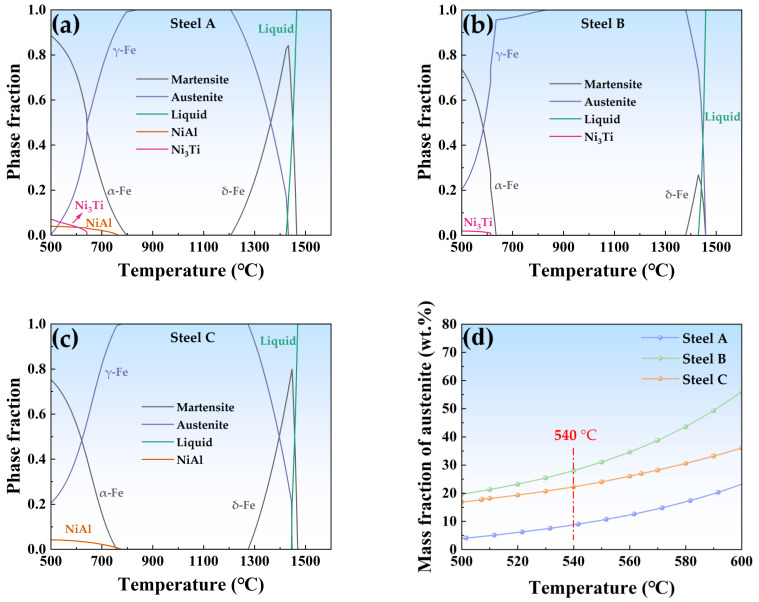
Thermodynamic calculation results of experimental steels: (**a**) Variation of phase fractions in the temperature range of (**a**) Steel A, (**b**) Steel B, and (**c**) Steel C; (**d**) Mass fraction of austenite in experimental steels versus temperature.

Thermo-Calc 2023a software with the TCFE9 database was used for thermodynamic calculations of the experimental steels (Figure 4). Figure 4a–c show that within the temperature range of 500–800 °C, the equilibrium microstructures of the three alloys contained strengthening phases and γ-Fe (austenite) in addition to α-Fe (martensite). The strengthening phases were Ni_3_Ti and NiAl in Steel A, Ni_3_Ti in Steel B, and NiAl in Steel C. This indicated that the type of strengthening phase was closely related to the presence of Al and Ti in the alloys.

The relationship between the mass fraction of austenite and temperature for the three alloys was calculated. As depicted in Figure 4d, at 540 °C, the mass fractions of austenite in Steel A, Steel B, and Steel C were 8.9%, 28.0%, and 22.2%, respectively. Under the same temperature, Steel B had the highest content of austenite.

### 3.2. XRD

**Figure 5:**
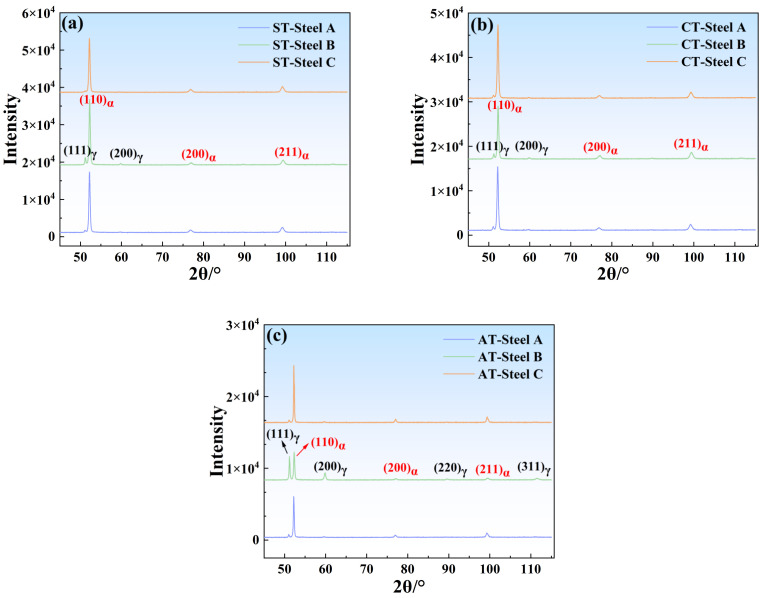
XRD patterns of experimental steels. Samples of (**a**) solution treatment; (**b**) cryogenic treatment; (**c**) aging treatment.

To determine the actual content of austenite in the steel, XRD was conducted on the three alloys in their solution-treated, cryogenic-treated, and aging-treated states, as shown in Figure 5. Figure 5a,b presents the XRD patterns of the alloys after solution treatment and cryogenic treatment, where Steel B contained austenite peaks of (111)γ, (200)γ, and (311)γ; however, in the diffraction patterns of Steel A and Steel C, the (200)γ and (311)γ peaks of austenite were vanishingly weak. Figure 5c displays the XRD patterns of the three alloys after aging treatment, wherein the XRD pattern of Steel B shows that the austenite peaks were (111)γ, (200)γ, (220)γ, and (311)γ, with the (111)γ and (200)γ peaks being significantly higher than those in Steel A and Steel C, and the (311)γ peak of austenite was not observed in the XRD patterns of Steel A and Steel C. Based on the aforementioned XRD patterns, the volume fraction of austenite was calculated using the quantitative phase percentage formula, Formula (1), and the results are listed in Table 3.

(1) $$V_{i}=\frac{\frac{1}{n}\sum_{i=1}^{n}\frac{I_{i}^{j}}{R_{i}^{j}}}{\frac{1}{n}\sum_{j=1}^{n}\frac{I_{\alpha }^{j}}{R_{\alpha }^{j}}+\frac{1}{n}\sum_{j=1}^{n}\frac{I_{\gamma }^{j}}{R_{\gamma }^{j}}},$$

In the formula, *i* represents the α and γ phases; *n* is the number of diffraction peaks of phase *i*; $R_{i}^{j}$*j* is the material scattering coefficient; $I_{i}^{j}$*j* is the integrated intensity of the diffraction peak, the value of which is the area of the diffraction peak; and *V_i_* is the volume fraction of phase *i*.

Table 3 shows that the volume fraction of austenite in the solution-treated state of Steel B was the highest at 12.40% while Steel A and Steel C were 3.85% and 3.05%, respectively. This result corresponds well with the Ms temperatures of the three alloys shown in Figure 3, indicating that steel samples with a lower Ms temperature contained more retained austenite. As demonstrated in Table 3, the retained austenite content in all three alloys decreased after cryogenic treatment compared to the solution-treated state but there was still 8.24% retained austenite in Steel B. After the aging treatment at 540 °C, the austenite content in all three alloys increased to varying degrees, indicating the formation of reversed austenite. At this point, the volume fractions of austenite in Steel A, Steel B, and Steel C were 8.67%, 53.66%, and 11.23% respectively. Steel B in the aged state also contained the highest volume fraction of austenite, which is in good agreement with the thermodynamic calculations presented earlier (Figure 4d), showing that Steel B has the highest austenite content at the same temperature.

### 3.3. Mechanical Properties

Table 4 presents the room-temperature tensile properties and impact toughness of the three alloys. The tensile data for the solution-treated state reveal that Steel A had the highest ultimate tensile strength (927 MPa) while Steel B had the lowest strength (836 MPa). The yield strength of Steel B was 111 MPa lower than that of Steel A. In conjunction with the data in Table 4, it is evident that the highest retained austenite content in the solution-treated Steel B was the primary reason for its lower tensile and yield strengths compared to Steel A and Steel C. As shown in Table 4, the UTS of solution-treated Steel A was higher than that of Steel C but its yield strength was lower. This was likely due to the effects of solid solution strengthening. In Steel A, the presence of 0.51Ti (wt.%) and 1.68Al (wt.%) resulted in more effective solid solution strengthening, hence a higher strength. However, the higher retained austenite content in solution-treated Steel A also led to a lower yield strength compared with that of Steel C, suggesting that austenite was a significant factor affecting the yield strength. Additionally, after aging treatment, the e_u_ and e_f_ of all three alloys decreased but the decline in Steel B was not significant. With an impact absorption energy of 209 J, Steel B demonstrated higher ductility and toughness, which can be attributed to the presence of 53.66% austenite after aging treatment, albeit with the smallest increase in strength. Similarly, aging-treated-state Steel C contained 11.23% austenite and, although its strength increase was less than that of Steel A, its impact absorption energy KU_2_ was twice that of Steel A, benefiting from the positive effect of austenite on its ductility and toughness. In summary, under the same conditions, Steel C showed better strengthening and toughness effects.

Notably, by comparison to PH13-8Mo and Custom 465, shown in Table 1, all of the three experimental alloys demonstrate superior toughness. Steel A, with an impact energy absorption of 50 J, surpasses Custom 465, yet lags behind Steel B, which records the highest value at 209 J, surpassing both PH13-8Mo (41 J) and Custom 465 (37 J). This increased toughness in Steel B is attributed to its elevated austenite content of 53.66%. Steel C, with an impact energy absorption of 115 J, more than doubles that of Custom 465 but is less than Steel B, indicating a synergistic contribution of austenite and strengthening phases to its toughness. These findings imply that the cobalt-free maraging stainless steels are well-suited for applications demanding high strength and toughness.

**Figure 6:**
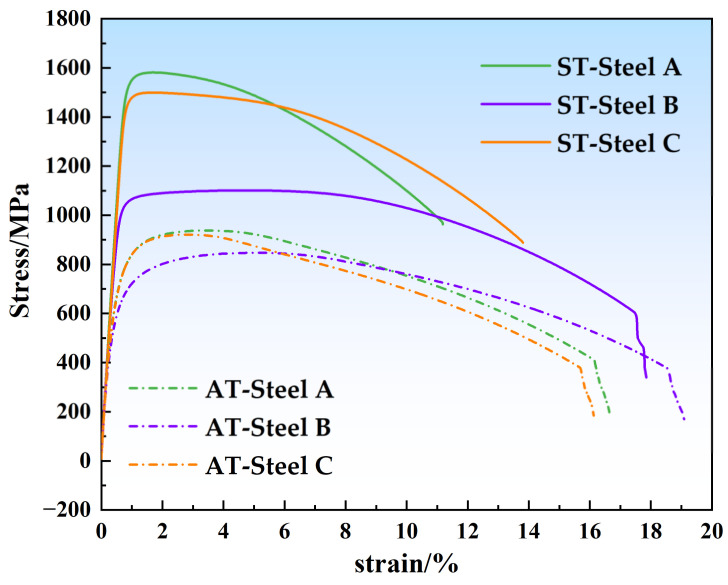
Stress–strain curves of experimental steels.

Figure 6 shows the stress–strain curves of the three alloys, as presented in Table 4. A comparison to the data in Table 4 reveals that after aging treatment, the strength of all three alloys increased, with Steel A showing the greatest increase, with a 644 MPa increase in its tensile strength and an 810 MPa increase in its yield strength. Steel C followed with the next-highest strength increase while Steel B had the smallest increase, with an increase of 260 MPa in its tensile strength and 409 MPa in its yield strength. This indicates that Steel A had the best aging strengthening effect, likely due to the combined action of the strengthening elements Ti and Al.

### 3.4. Fractography

**Figure 7:**
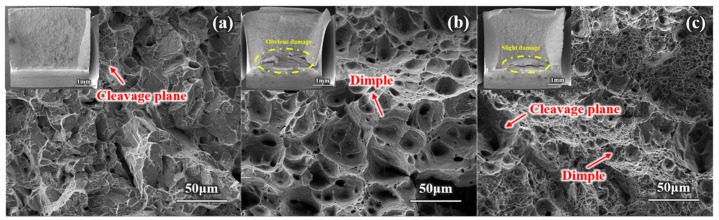
SEM images of the impact fracture surface of the experimental steels: (**a**) Steel A, (**b**) Steel B, and (**c**) Steel C.

Figure 7 presents the fracture morphologies of aged impact specimens, as observed by SEM. Figure 7a shows that the impact fracture morphology of Steel A exhibited a typical sugar-like pattern, with the fracture mode being an intergranular brittle fracture (Figure 7a). Figure 7b shows that the impact fracture of Steel B was composed of a fibrous zone and a shear lip area and the fracture underwent some contraction perpendicular to the direction of impact stress, with an impact energy absorption of 209 J, indicating a ductile fracture. In the fibrous zone of the impact fracture of Steel C, both cleavage steps and dimples were observed (Figure 7c), indicating a mixed fracture mode of ductility and brittleness. In summary, the fracture morphology corresponded well with the impact energy absorption of the three alloys.

### 3.5. Microstructure

**Figure 8:**
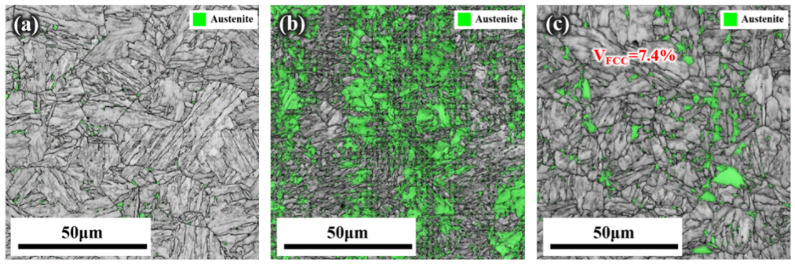
EBSD results of experimental steels. Band contrast (BC) images of (**a**) Steel A sample, (**b**) Steel B sample, and (**c**) Steel C samples after aging treatment.

Figure 8 illustrates the EBSD results of the three alloys in their aged conditions, with the green areas representing austenite. As shown in Figure 8a, after aging, austenite in Steel A was distributed along the original austenite grain boundaries and lath boundaries. Steel C exhibited a similar austenite distribution, but significantly more than that of Steel A (Figure 8c). As depicted in Figure 8b, Steel B contained the most austenite, which was also the main reason for its high-impact energy absorption and retained ductility.

**Figure 9:**
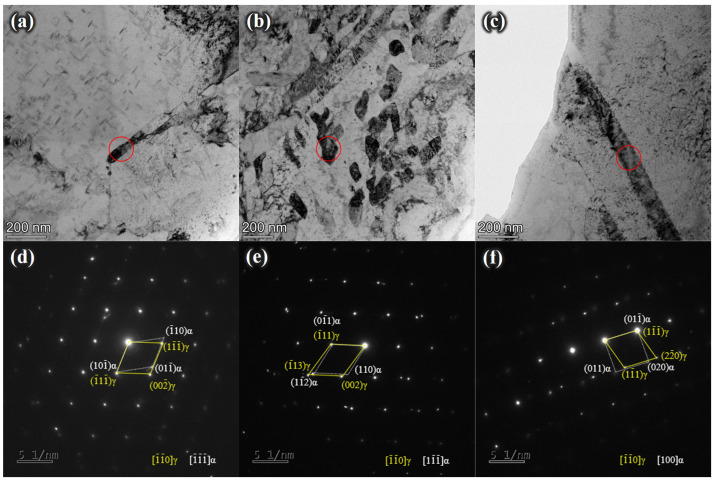
TEM micrographs of experimental steels: (**a**–**c**) bright-field images and (**d**–**f**) the corresponding diffraction spots of (**a**,**d**) Steel A, (**b**,**e**) Steel B, and (**c**,**f**) Steel C.

As shown in Figure 9a,c, austenite in Steel A was distributed along the original austenite grain boundaries and lath boundaries, with a similar distribution observed in Steel C but with much more austenite than in Steel A. Figure 9b illustrates that Steel B contained the most austenite, which is also the main reason for its higher-impact energy absorption and retained ductility. Normalizing the diffraction spots (Figure 9d–f) showed a Kurdjumov–Sachs (K–S) orientation relationship between austenite and martensite in Steel A and Steel B and a Nishiyama–Wassermann (N–W) orientation relationship in Steel B. This suggests that as austenite gradually grew from a thin film to a blocky shape, its orientation relationship with martensite changed [22,23].

**Figure 10:**
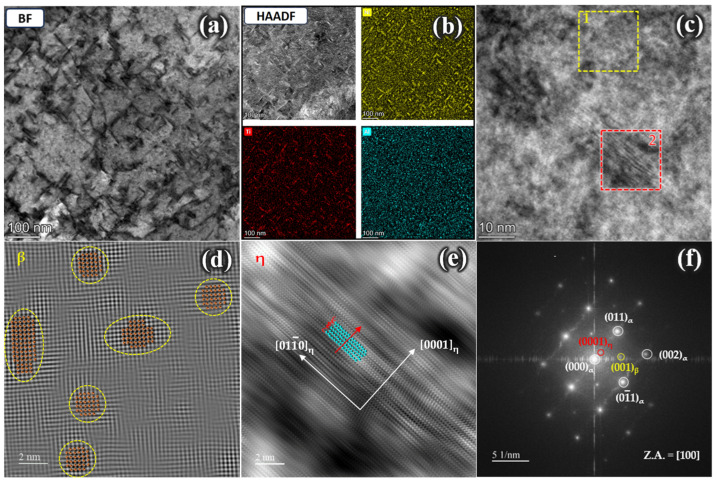
Scanning transmission electron microscopy (STEM) images of Steel A: (**a**) bright-field (BF) image revealing high-density nanoprecipitates distributed within the martensitic matrix; (**b**) the corresponding high-angle annular dark-field (HAADF) image and EDS element maps of (**a**); (**c**) high-resolution transmission electron microscopy (HRTEM) image; (**d**,**e**) atomic-resolution HAADF-STEM images of the β and η phases from Square Areas 1 and 2 in (**c**); (**f**) fast Fourier transformation (FFT) of (**c**).

Figure 10 displays the STEM micrographs of the strengthening phases observed in aged Steel A. The bright-field image in Figure 10a shows many finely dispersed strengthening phases throughout the matrix. Energy-dispersive spectroscopy (EDS) maps of Ni, Ti, and Al in this area (Figure 10b) indicated that the strengthening phases exhibited both rod-like and spherical morphologies. Figure 10c shows a magnified area, with Positions 1 and 2 marked for different morphologies, which were subjected to high-resolution analysis. The results indicate that the spherical precipitates at Position 1 were β-NiAl and the rod-like precipitates at Position 2 were η-Ni_3_Ti. Figure 10d (corresponding to Position 1) shows a high-resolution image of the spherical β-NiAl phase with an ordered B2 structure. No distinct particle/matrix interface was observed, suggesting a high degree of coherence between the precipitate and the matrix. Figure 10e (corresponding to Position 2) presents a high-resolution image of the rod-like η-Ni_3_Ti phase. Figure 10f shows a selected area electron diffraction (SAED) pattern obtained via fast Fourier transform (FFT), confirming that Steel A contained two types of nanoscale precipitates, namely, β-NiAl and η-Ni_3_Ti.

**Figure 11:**
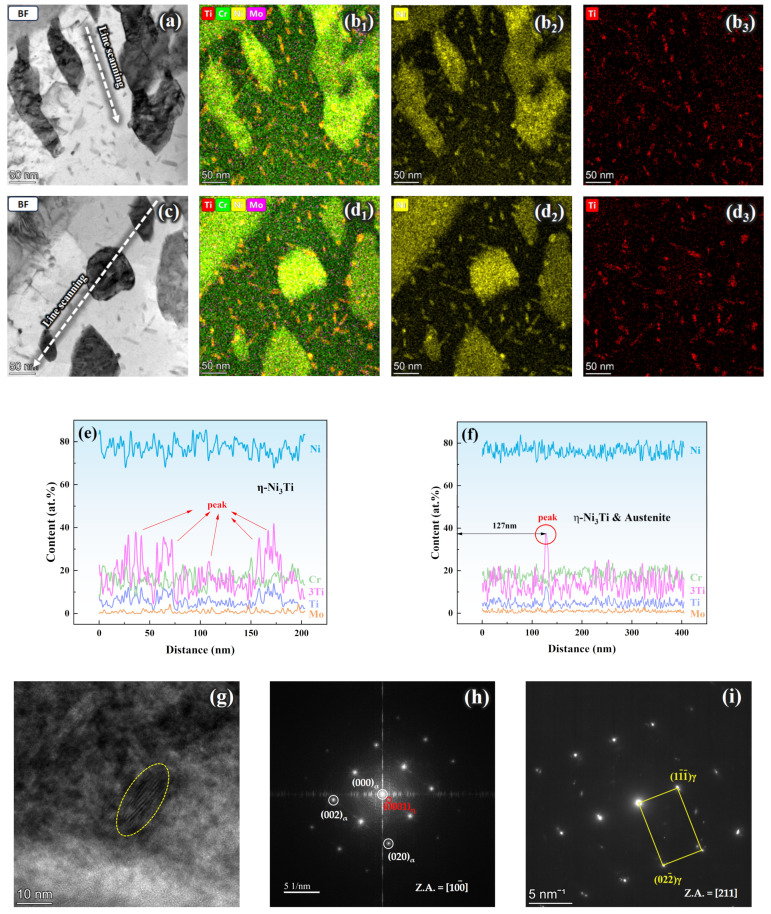
STEM images of austenite and precipitates in Steel B: (**a**,**c**) bright-field (BF) image; (**b_1_**–**b_3_**) EDS element map of (**a**); (**d_1_**–**d_3_**) EDS element map of (**c**); (**e**) chemical distribution along the white dashed line in (**a**); (**f**) chemical distribution along the white dashed line in (**d**); (**g**) HRTEM image; (**h**) FFT results of (**g**); (**i**) corresponding diffraction spot of the blocky precipitate in (**c**).

Figure 11 presents STEM micrographs of the matrix of Steel B after the aging treatment. Figure 11a and Figure 11c are bright-field images, revealing the presence of blocky precipitates and rod-shaped phases in Steel B. The EDS element maps in Figure 11b_1_–b_3_ and Figure 11d_1_–d_3_ indicate that the rod-shaped phases were rich with Ni and Ti while the blocky precipitates were Ni-enriched. Figure 11g shows a high-resolution image of the rod-shaped precipitate and its corresponding diffraction spots, confirming that the rod-shaped phase in Steel B was also η-Ni_3_Ti. Figure 11i displays diffraction spots corresponding to the blocky precipitate and the calibration results identified it as austenite. The line scans for Figure 11a,c are shown in Figure 11e,f, respectively. The pink 3Ti (at.%) line corresponds to the atomic percentage of Ni in η-Ni_3_Ti and the blue line represents the total atomic percentage of Ni. As shown in Figure 11a, when the line scan passed only through the rod-shaped precipitate, the 3Ti line exhibited multiple peaks corresponding to the location of the η-Ni_3_Ti phase. The 3Ti line was close to the Ni (at.%) line, indicating that Ni was primarily consumed for the formation of the η-Ni_3_Ti precipitate. When the line scan passed through both austenite and the rod-shaped precipitate (Figure 11c), a noticeable peak in the 3Ti line was observed at 127 nm. Combined with the EDS results in Figure 11d_1_–d_3_, it can be concluded that this peak corresponded to the interface between Ni_3_Ti and austenite. The lower position of the 3Ti line, which was farther from the Ni line, suggests that a significant amount of Ni was consumed by austenite. These results demonstrated that the substantial amount of blocky reversed austenite and the sole strengthening phase Ni_3_Ti in Steel B competed for Ni, which was supported by the relevant literature [24,25,26,27,28]. The aforementioned analysis reveals that the strengthening phase in Steel B was η-Ni_3_Ti, accompanied by a large amount of blocky austenite rich in Ni.

**Figure 12:**
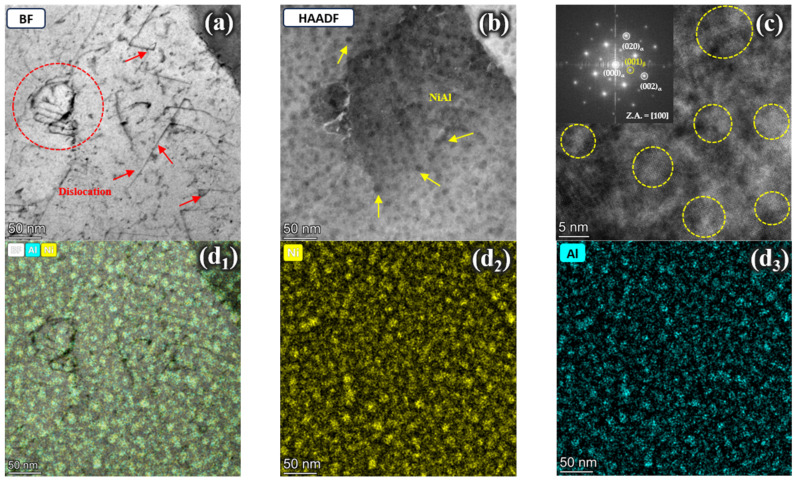
STEM images of Steel C: (**a**,**b**) bright-field (BF) and HAADF images showing the dislocation-precipitate interaction; (**c**) HRTEM images and the corresponding fast Fourier transformation (FFT) results; (**d_1_**–**d_3_**) EDS element maps.

Figure 12 presents STEM micrographs of the matrix in Steel C after aging treatment. Figure 12a,b show many dispersed, fine spherical strengthening phases within the matrix with a high dislocation density. As shown in Figure 12d_1_–d_3_, the EDS element maps of the area revealed that the spherical precipitates were rich in Ni and Al. Combined with the diffraction spot calibration result in Figure 12c, it can be determined that the spherical precipitates were β-NiAl phases. The image shows a multitude of spherical NiAl nanoprecipitates exhibited strong interactions with dislocations, demonstrating a significant pinning effect on the dislocation motion, thereby maximizing the strength of Steel C. However, no blocky austenite or rod-shaped precipitates were observed in the STEM images, indicating that no η phase precipitated during the aging process of Steel C.

## 4. Discussion

**Figure 13:**
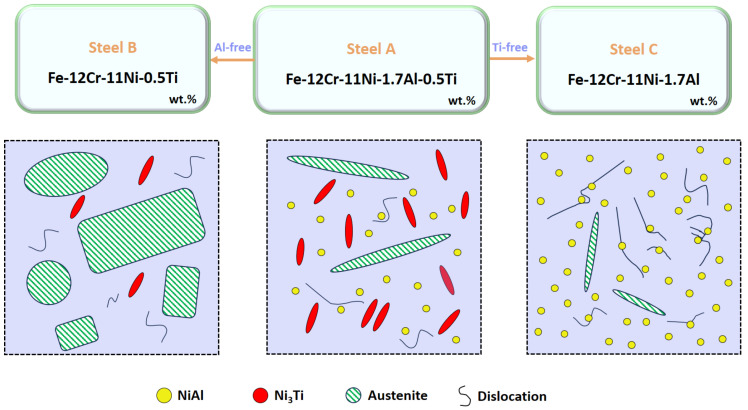
Illustration of the role of Al/Ti in the microstructure after the aging treatment.

This study presents a detailed investigation into the role of Al and Ti in precipitate-strengthened and austenite-toughened Co-free maraging stainless steels. Through a meticulous examination of three specific alloys—Steel A, Steel B, and Steel C—we have gained valuable insights into the microstructural evolution and mechanical properties in accordance with the established heat treatment process. Figure 13 provides a description of the microstructures of the three alloys after aging treatment. Considering the composition and mechanical properties of the three alloys under the same heat treatment state, Steel A exhibited the highest strength while Steel B showed the lowest. In Steel A, Ti and Al have a strong solution effect and two types of strengthening phases, including spherical β-NiAl and rod-shaped η-Ni_3_Ti, were precipitated during the aging treatment. The M_s_ temperature of Steel B was the lowest due to a lack of Al, resulting in the highest retained austenite content after the solution treatment. Although cryogenic treatment reduced the austenite content to 8.24%, aging at 540 °C significantly increased the Ni-rich austenite content to 53.66%, along with the precipitation of the η-Ni_3_Ti phases. The competition for Ni atoms between the reversed austenite and the strengthening phases resulted in a reduced quantity of η-Ni_3_Ti, limiting the strength increase of Steel B. After the aging treatment, the strength of Steel C increased by 584 MPa, attributable to the nucleation of a multitude of dispersed, coherent, spherical β-NiAl precipitates within a matrix characterized by a high dislocation density. These phases effectively pinned dislocations hindering their motion, thus maximizing the aging strengthening effect. Under this condition, the austenite content in Steel C was 11.23% and the impact absorption energy was 115 J, more than double that of Steel A. This indicates that Al has a more favorable strengthening and toughening effect on the steel and Steel C demonstrates the best balance of strength and toughness.

It is important to note that our study is limited to a specific set of alloys and, thus, the generalizability of these findings to other maraging stainless steel compositions may be limited. The strengthening and toughening mechanisms of Al and Ti have been explored, yet a more nuanced understanding of the microstructure–property relationships is warranted. This includes a detailed characterization of the precipitate sizes, distributions, and volume fractions, which are critical parameters in determining the mechanical properties of these alloys. The heat treatment conditions employed in this study, while carefully controlled, may not encapsulate the full spectrum of processing conditions that could influence the alloys’ properties. Therefore, exploring a broader range of heat treatment parameters could reveal additional insights into the optimization of these alloys.

Looking ahead, several avenues of research present themselves as promising for further exploration. We could broader alloy systems expanding the scope of this study to include a wider range of Co-free maraging stainless steels with varying concentrations of Al, Ti, and other alloying elements. Therefore, a more comprehensive understanding of the effects of these elements on mechanical properties would be provided. Additionally, utilizing advanced microscopy techniques, such as Three-Dimensional Atom Probe tomography (3D-APT), and computational modeling could offer detailed insights into the precipitate sizes, distributions, and their interactions with dislocations, thereby enhancing our understanding of the strengthening mechanisms. Investigating the long-term stability of the precipitates and austenite phase under various service conditions is essential for predicting the service life and reliability of these alloys, especially in demanding applications, such as aerospace and nuclear industries.

By addressing these limitations and exploring the proposed future directions, the research on Co-free maraging stainless steels can be advanced, leading to the development of superior materials for various engineering applications. This study would contribute to the growing body of knowledge on high-strength stainless steels and pave the way for innovative solutions in material design and engineering.

## 5. Conclusions

From the aforementioned research, it is evident that in the Fe-12Cr-11Ni-1.7Al-0.5Ti alloy, Ti and Al exhibit distinct strengthening and toughening effects, leading to the following conclusions:

(1) In the Fe-12Cr-11Ni-1.7Al-0.5Ti alloy, Ti and Al demonstrate significant solid solution effects, with the tensile strength of the solution-treated state reaching 927 MPa. Subsequent aging treatment leads to the simultaneous precipitation of spherical β-NiAl and rod-like η-Ni_3_Ti phases, increasing the tensile strength to 1571 MPa;
(2) In the Fe-12Cr-11Ni-0.5Ti alloy, the absence of the Al lowers the Ms temperature, resulting in the highest amount of austenite after solution treatment. Although cryogenic treatment can reduce its quantity, aging at 540 °C increases the austenite content to 53.66%. The Ni-rich reversed austenite sequesters Ni atoms that could otherwise precipitate more η-Ni_3_Ti phases, leading to a modest increase in strength. This observation diverges from the work of Leitner et al. [20], who observed an increase in strength with Ti addition due to the formation of η-Ni_3_Ti without the competing influence of austenite;
(3) The Fe-12Cr-11Ni-1.7Al alloy shows the best balance of strength and toughness. Al not only contributes to solid solution strengthening but also leads to the precipitation of coherent spherical β-NiAl phases around a high dislocation density matrix during aging treatment. These phases are finely and sparsely distributed in the matrix, which is the primary reason for the tensile strength of 1492 MPa. This finding aligns with prior research by Charline et al. [10], who reported the beneficial effects of Al on strengthening via the precipitation of β-NiAl. At this point, the austenite content in the alloy is 11.23% and the impact absorption energy is 115 J, more than double that of the Fe-12Cr-11Ni-1.7Al-0.5Ti alloy. Our findings are corroborated by studies, such as those by Zhou et al. [23], who reported the beneficial effects of Al on the stability of austenite and the overall toughness of maraging steels.

In summary, our research provides a comprehensive analysis of the strengthening and toughening mechanisms of Al and Ti in Co-free maraging stainless steels. By comparing our results with the existing literature, it is evident that the optimized use of Al and Ti can significantly enhance the performance of the Fe-12Cr-11Ni-1.7Al-0.5Ti alloy. Our findings not only validate previous research on the individual effects of Al and Ti but also offer new insights into their combined action, particularly in the context of austenite formation and its impact on mechanical properties. This study, therefore, contributes to the ongoing efforts in developing high-strength, cost-effective stainless steels for demanding applications in various industries.

## Figures and Tables

**Table 1:** Chemical composition, strengthening phase, and mechanical properties of typical Co-free maraging steels [9,10,11,12,13,14].

Steel	Mass Fraction (%)						Strengthening Phase	UTS (MPa)	KU_2_ (J)
	C	Ni	Cr	Mo	Ti	Al			
PH13-8Mo	0.03	8.2	12.7	2.2	-	1.1	NiAl	1551	41
Custom465	0.02	10.9	11.6	1.0	1.5		Ni_3_Ti	1779	37
MLX17	0.02	11.25	12.5	2.0	0.5	1.75	NiAl/Ni_3_Ti	1725	44
MLX19	0.02	13.0	10.5	2.0	1.5	1.7	NiAl/Ni_3_Ti	1895	26

UTS = ultimate tensile strength, KU_2_ = U-notch impact absorption energy.

**Table 2:** Chemical composition of experimental steels (wt.%).

Steel	C	Cr	Mo	Ni	Ti	Al
Steel A	0.0010	11.71	2.10	10.89	0.51	1.67
Steel B	0.0009	11.65	2.10	10.94	0.43	-
Steel C	0.0010	11.77	2.09	10.81	-	1.70

**Table 3:** Austenite in experimental steels (%).

Steel	Volume Fraction of Austenite (%)	Steel	Volume Fraction of Austenite (%)	Steel	Volume Fraction of Austenite (%)
ST-Steel A	3.85	CT-Steel A	3.02	AT-Steel A	8.67
ST-Steel B	12.40	CT-Steel B	8.24	AT-Steel B	53.66
ST-Steel C	3.05	CT-Steel C	2.78	AT-Steel C	11.23

**Table 4:** Mechanical properties of experimental steels.

Steel	UTS (MPa)	YS(0.2% Off-Set) (MPa)	KU_2_ (J)	e_u_ (%)	e_f_ (%)
AT-Steel A	1571	1529	50	12.5	65
AT-Steel B	1096	1017	209	18.0	76
AT-Steel C	1492	1460	115	13.8	66
ST-Steel A	927	719	-	16.5	78
ST-Steel B	836	608	-	19.5	80
ST-Steel C	908	724	-	16.0	80

## Data Availability

The raw/processed data required to reproduce these findings cannot be shared at this time as the data also form part of an ongoing study.
